# AIDS mortality in African migrants living in Portugal: evidence of large social inequalities

**DOI:** 10.1136/sti.2008.034066

**Published:** 2009-06-11

**Authors:** L M Williamson, M Rosato, A Teyhan, P Santana, S Harding

**Affiliations:** 1 Medical Research Council Social and Public Health Sciences Unit, Glasgow, UK; 2 Centre for Public Health, Queen’s University, Belfast, UK; 3 Centro de Estudos de Geografia e Ordenamento do Território, Universidade de Coimbra, Coimbra, Portugal

## Abstract

**Objective:**

To examine infectious disease and AIDS mortality among African migrants in Portugal, gender and socio-economic differences in AIDS mortality risk, and differences between African migrants to Portugal and to England and Wales.

**Methods:**

Data from death registrations, 1998–2002, and the 2001 Census were used to derive standardised death rates by country of birth, occupational class (men only), and marital status.

**Results:**

Compared with people born in Portugal, African migrants had higher mortality for infectious diseases including AIDS. There was considerable heterogeneity among Africans, with those from Cape Verde having the highest mortality. Death rates were more than five times higher among those who were unmarried than those who were. A larger proportion of Africans were unmarried accounting for some excess mortality. Death rates were also higher among men from manual occupational classes than among men from non-manual. A comparison with England and Wales shows that death rates for infectious disease and AIDS in Portugal are much higher and Africans in Portugal also fare worse than Africans in England and Wales.

**Conclusion:**

AIDS mortality rates were higher among Africans than those born in Portugal and were associated with socio-environmental factors. Further research is required to interpret the excess mortality among Africans and there is a need to ensure the inclusion of relevant data items on ethnicity in national monitoring and surveillance systems.

HIV/AIDS is a major public health concern for Western Europe and migrant populations fare worse than the indigenous populations of receiving countries with individuals originating from countries with generalised epidemics accounting for a disproportionate number of new heterosexually acquired infections.^1^ However, interpretation of this is hindered by often inadequate and incomplete data on ethnicity,^2^ ^3^ which has the unfortunate effect of assuming homogeneity of disease experience across and within ethnic communities.

Reductions in mortality in Portugal mean that the health of its population is now comparable to that of other countries in Western Europe,^4^ with an adult mortality rate of 93 per 1000 (compared to 80 per 1000 in the UK) and life expectancy of 79 years (the same as in the UK).^5^ However, although AIDS mortality has fallen dramatically in Western Europe since the introduction of antiretroviral treatments in the mid-1990s, rates remain high in Portugal (66/million in 2006 compared with an average of 15.9/million in Western Europe).^1^ Portugal also has the highest HIV infection rates (205/million in 2006 compared with an average of 82.5/million for Western Europe and 149/million for the UK).^1^ Injecting drug use (IDU) has historically been the predominant mode of transmission but heterosexual transmission has recently reached comparable levels.^6^ ^7^ Portugal is one of the main receiving countries for African migrants in the south of the European Union, with most originating from its ex-colonies, principally Cape Verde, Angola and Mozambique. Portugal has large social inequalities in health,^8^ and research has shown high AIDS mortality rates in the urban areas of Greater Lisbon, which have large African migrant populations,^7^ but differences in mortality rates between African migrants and those born in Portugal have not been explored. In this paper, we compare the infectious disease and AIDS mortality rates of African migrants with those born in Portugal and examine gender and socio-economic differences in AIDS mortality risk. Comparisons are also made with African migrants to England and Wales.

## Methods

The Instituto Nacional de Estatística in Portugal provided anonymised death records for 1998–2002 and tabulated population data from the 2001 Census. Deaths and populations-at-risk were derived by country of birth and 5-year age groups with analyses based on those aged 25–64 years. This age restriction was because of small numbers of deaths at younger ages and the potentially poor quality of denominator data at older ages due to return migration to home countries. We identified people born in Portugal (n = 5 093 910), referred to hereafter as Portugal-born, and those born in African countries (Angola = 140 770, Mozambique = 64 927, Cape Verde = 32 871, Guinea = 235, Equatorial Guinea = 50, Guinea Bissau = 14 871, St Tome and Principe = 8246), referred to hereafter as Africans. Marital status was classified as unmarried or married, and occupational class as non-manual, manual or unclassified (if information on occupation was inadequate or missing). Two tables containing denominators from the census were supplied: one with age by country of birth, gender and marital status, and another with age by country of birth, gender and occupational class. A single table with marital categories within occupational classes was not allowed because of its potential to identify individuals precluding analysis of the joint effect of these variables on mortality. Analysis by occupational class was limited to men as a large number of deaths among women were not classified.

Death rates for all infectious diseases, as well as AIDS, were derived because of the possibility of misclassification of AIDS deaths (recognising that the infectious diseases tuberculosis and pneumonia are the most commonly reported AIDS-defining illnesses in Western Europe^1^). Death rates from pneumonia were not provided separately due to the potential identification of individuals. The 9th International Classification of Disease was used to classify deaths occurring between 1998 and 2000 (codes 000–139 for all infectious disease; 042–044 for AIDS) and the 10th for deaths between 2001 and 2002 (codes A00–A98 and B00–B99 for all infectious disease; B20–B24 for AIDS). Directly standardised rates for both the African migrants and Portugal-born adjusted to the European standard population 2000, rate ratios (RRs) using the rate of the Portuguese as a reference rate and 95% confidence intervals (CIs) were derived.^9^ ^10^ Rates were derived for all Africans and for the three largest groups (Angola, Mozambique and Cape Verde). Where at least 20 deaths were recorded in each 10-year age group, poisson regression models were used to examine the contribution of occupational class and (separately) marital status to the mortality differences between African migrants and Portugal-born.

Similar data for African migrants to England and Wales were used to derive corresponding death rates and RRs, which enabled comparisons with Africans in Portugal. The Office for National Statistics provided anonymised death records for 1999–2003 and tabulated population data from the 2001 census for England and Wales. Deaths and populations at risk were again derived by country of birth and 5-year age groups with analyses based on those aged 25–64 years only. The UK comparison group contained those born in countries from Western and Southern Africa (termed West Africa hereafter) that were least likely to have significant white Africans (according to 2001 distributions of ethnicity by country of birth). These were Gambia, Ghana, Sierra Leone, Nigeria, Botswana, Lesotho, Swaziland and Zimbabwe.

## Results

**Figure 1:**
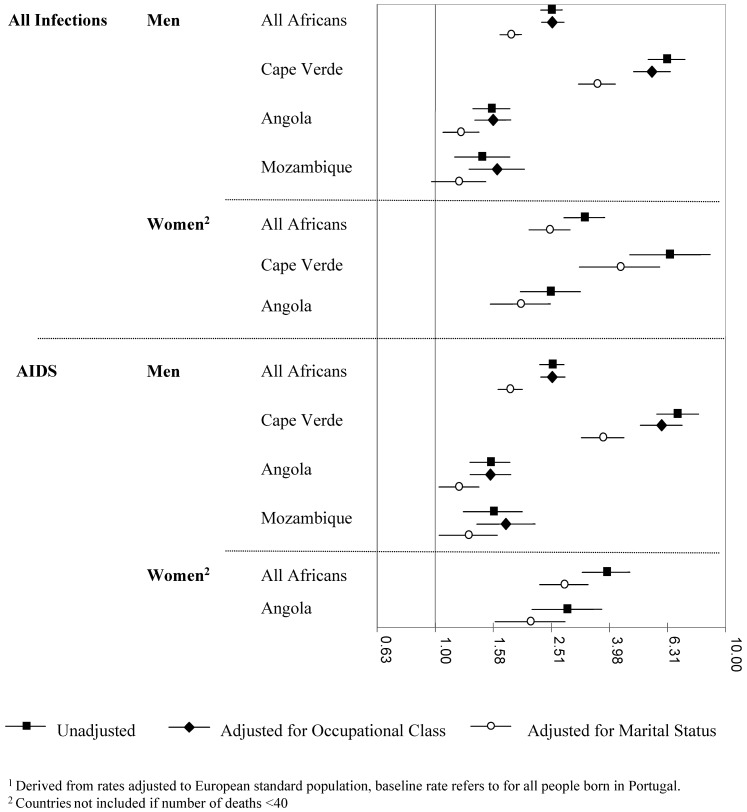
The impact of adjusting for marital status and for occupational class on infectious diseases and AIDS mortality of African migrants living in Portugal. RR and 95% CI, ages 25–64 years, period 1998–2002. 1Derived from rates adjusted to European standard population, baseline rate refers to for all people born in Portugal. 2Countries not included if number of deaths <40.

Among Portugal-born, infectious diseases accounted for 5.7% of all deaths (of which 67.6% were AIDS and 9.7% were tuberculosis). The corresponding figures for Africans were 16.8%, 79.3% and 7.7%. Death rates for both all infectious diseases and AIDS were higher among Africans than Portuguese (fig 1), with death rates for AIDS among men from Cape Verde men more than twice that of Portugal-born men.

Adjustment for differences in the distributions of marital status categories reduced some of the excess mortality for both all infectious diseases and AIDS, more so among men than women. In both Portuguese and Africans, those who were not married had substantially higher death rates than those who were married (table 1). Among men, the relative difference was greater among Portugal-born than Africans. However, the absolute level of mortality was greater in Africans than Portugal-born in every marital status category. Occupational class was also associated with all infectious diseases and AIDS mortality among men. Men in manual social classes had higher death rates than men in non-manual, but the relative difference, as reflected by the RRs, appeared to be smaller than for marital status.

**Table 1:** Death rates* and RR†, 95% CI, by marital status and by occupational class (men) for African migrant and Portugal-born men and women, ages 25–64 years

	Portugal born		African migrant		RR†‡ (95% CI)
	Deaths	Rate/RR (95% CI)	Deaths	Rate/RR (95% CI)	
a. Men					
All infections					
Non-manual	340	10.9 (9.7 to 12.1)	49	29.5 (18.9 to 40.1)	2.71 (1.86 to 3.95)
Manual	1893	27.4 (26.1 to 28.7)	319	67.7 (47.0 to 88.5)	2.47 (2.11 to 2.88)
Unclassified	2020	115.7 (110.3 to 121.1)	241	255.2 (221.0 to 289.5)	2.21 (1.92 to 2.55)
RR manual vs non-manual		2.51 (2.23 to 2.83)		2.29 (1.55 to 3.38)	
Married	1275	13.1 (12.4 to 13.8)	125	43.1 (34.7 to 51.4)	3.29 (2.69 to 4.02)
Not married	2971	101.9 (97.8 to 106.0)	484	233.6 (204.4 to 262.8)	2.29 (2.01 to 2.61)
RR not married vs married		7.78 (7.26 to 8.34)		5.42 (4.34 to 6.76)	
Overall rate	4253	33.9 (32.9 to 34.9)	609	108.8 (98.5 to 119.1)	3.21 (2.91 to 3.55)
AIDS					
Non-manual	223	6.6 (5.7 to 7.5)	41	22.2 (13.4 to 31.0)	3.36 (2.21 to 5.10)
Manual	1394	19.3 (18.2 to 20.3)	246	92.3 (76.6 to 108.0)	4.78 (4.00 to 5.71)
Unclassified	1392	89.4 (84.6 to 94.3)	197	198.8 (169.3 to 228.4)	2.22 (1.89 to 2.60)
RR manual vs non-manual		2.92 (2.53 to 3.37)		4.16 (2.70 to 6.40)	
Married	620	6.7 (6.1 to 7.2)	92	30.3 (23.4 to 37.1)	4.52 (3.56 to 5.75)
Not married	2382	74.5 (71.1 to 77.8)	392	176.5 (151.6 to 201.3)	2.37 (2.04 to 2.75)
RR not married vs married		11.12 (10.14 to 12.20)		5.83 (4.52 to 7.53)	
Overall rate	3009	23.8 (23.0 to 24.7)	484	81.7 (73.0 to 90.4)	3.43 (3.07 to 3.84)
b. Women					
All infections					
Married	429	4.4 (3.9 to 4.8)	26	10.2 (5.9 to 14.6)	
Not married	622	18.7 (17.2 to 20.2)	148	56.2 (45.5 to 66.9)	3.01 (2.45 to 3.70)
RR not married vs married		4.25 (3.75 to 4.82)		5.51 (3.51 to 8.66)	
Overall rate	1056	8.0 (7.5 to 8.5)	174	29.5 (24.4 to 34.5)	3.69 (3.08 to 4.42)
AIDS					
Married	151	1.6 (1.3 to 1.8)	13	5.3 (2.1 to 8.5)	
Not married	424	12.7 (11.4 to 13.9)	124	45.5 (36. To 55.0)	3.58 (2.84 to 4.51)
RR not married vs married		7.94 (6.57 to 9.60)		8.58 (4.62 to 15.92)	
Overall rate	579	4.4 (4.1 to 4.8)	137	22.2 (17.9 to 26.5)	5.05 (4.09 to 6.22)

Portugal 1998–2002.

*Adjusted to European standard population; †rate for Portugal in same marital status or occupation class category  =  baseline; ‡RR missing where number of deaths insufficient.

**Figure 2:**
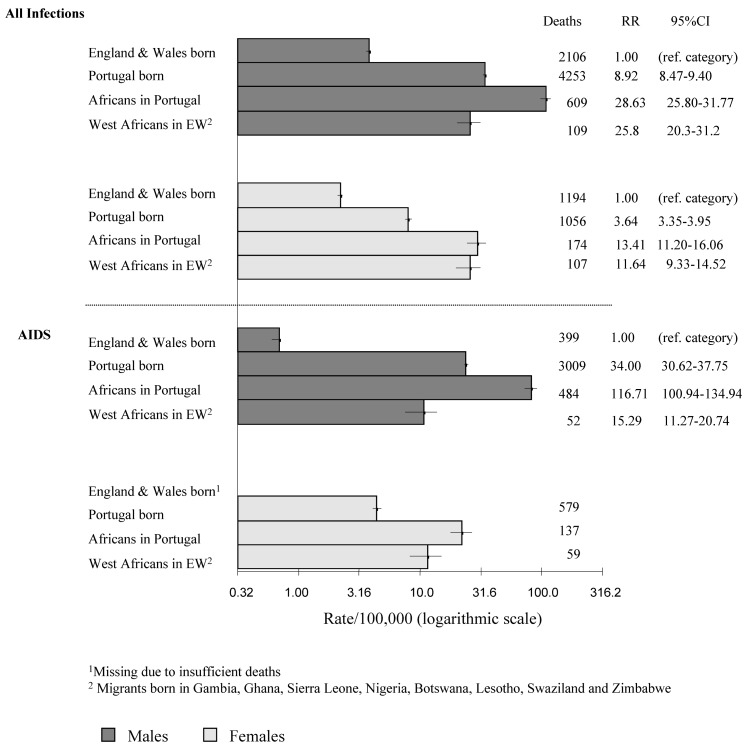
Death rates (adjusted to European standard population.), RR and 95% CI for African origin groups living in Portugal (1998–2002) or England and Wales (1999–2003), aged 25–64 years. 1Missing due to insufficient deaths. 2 Migrants born in Gambia, Ghana, Sierra Leone, Nigeria, Botswana, Lesotho, Swaziland and Zimbabwe.

Figure 2 shows the RRs for people born in Portugal, African migrants in Portugal and in England and Wales, using the death rates for people born in England and Wales as the reference rates. Infectious disease and AIDS death rates were much higher in Portugal than in England and Wales. Among men, AIDS death rates for those who were Portugal-born were more than 30 times higher and more than 100 times higher for Africans in Portugal, than the rates for men born in England and Wales. Africans in Portugal had higher AIDS death rates (men 87.7/100 000; women 22.2/100 000) than Africans in England and Wales (10.7/100 000; women 11.6/100 000). In the England and Wales grouping of West Africans, migrants from Zimbabwe comprised about 20% of the population but 76% of AIDS deaths.

## Discussion

To our knowledge, this is the first study to examine AIDS mortality among Africans in Portugal. Portugal has the highest HIV infection and AIDS rates in Western Europe,^1^ ^11^ and our findings show higher mortality rates for all infectious diseases and AIDS among African migrants than those born in Portugal. Infectious disease and AIDS death rates were higher among those who were unmarried than those who were, and among men from manual occupational classes than among non-manual.

A greater proportion of Africans were not married and this accounted for some of their excess mortality. It is possible that being unmarried is associated with greater exposure to HIV risk factors, such as having multiple sexual partners.^12^ The association between poverty, unemployment and HIV is also often cited as a key feature of the African migrant experience of the disease in Europe.^12^ However, we have found that African men in Portugal are more likely to be classified to both an occupational class (signalling economic activity) and a non-manual class than those born in Portugal,^7^ ^13^ and here we find that the effect of occupational class on mortality was similar for both Africans and Portugal-born. Although we could not examine the joint effects of marital status and occupational class, these results suggest that there are clear social inequalities in mortality risk of Africans, but the high risk cannot be attributed only to poverty and unemployment.

Although life expectancy in England and Wales and Portugal is very similar, our comparison shows higher mortality from infectious disease and AIDS in Portugal. This is perhaps not unexpected given that Portugal has the highest rates of HIV and AIDS in Western Europe.^1^ IDU remains the predominant mode of HIV transmission (46%), which distinguishes Portugal from the rest of Western Europe, but it is now closely followed by heterosexual transmission (43%).^6^ This may be evidence of the increase in the proportion of HIV cases found among migrants from sub-Saharan Africa throughout Western Europe,^6^ but whether IDU is also an issue in this population is unknown. However, Africans in Portugal also fare worse than Africans in England and Wales. This is the case even though migrants from sub-Saharan Africa account for a smaller proportion of new HIV infections in Portugal than in the UK (15% and 24%, respectively).^1^

West Africans in the UK are mainly from Nigeria and Ghana—countries that are poor compared with Western countries but which have not recently been disrupted by protracted civil wars or natural disasters (unlike Angola and Mozambique, home countries of African migrants in Portugal). Therefore, differential patterns in migration could be expected. Variation in the HIV prevalence rates of the home countries of Portugal and UK migrants could also affect susceptibility to risk. Mozambique has a much higher prevalence (12.5%) than Angola (2.1%), Nigeria (3.1%) or Ghana (1.9%).^14^ Recent migration to the UK from economically disrupted countries with high prevalence (such as Zimbabwe and Congo) may have had some impact on AIDS prevalence given the large contribution to the deaths from AIDS in the West African grouping.

Issues related to poor access to specialist care, and late presentation and HIV diagnosis have been highlighted as important in understanding African migrants’ experience of HIV/AIDS in Europe.^3^ ^15^ ^16^ ^17^ ^18^ ^19^ ^20^ In the UK in 2006, 41% of black Africans had a CD4 count below 200 cells/mm^3^ at HIV diagnosis (which at the time was the recommended threshold for treatment to commence) and 11% had an AIDS defining illness compared with 33% and 8%, respectively, in the overall population.^21^ Similar data from Portugal are currently unavailable but it is possible that late diagnosis accounts for some of the excess mortality among migrants. Although migrants are entitled to free primary health care and Portugal provides free antiretroviral treatment to HIV patients, including foreign citizens who legally reside there, studies of migrants in the Greater Lisbon area have found their use of primary healthcare services to be low.^20^ ^22^ Once receiving treatment and care, migrants’ disease progression and survival should not differ from indigenous populations and early diagnosis and timely access to treatment are essential to reduce AIDS mortality. Further research is required to examine whether these are issues for migrant populations in Portugal.

These findings are subject to the usual limitations of cross-sectional data— notably misclassification of country of birth between the census and death certificates, and the potential for confounding caused by health selection on migration. Higher proportions of African men were classified to both an occupational class and a non-manual class, suggesting some positive selection (more skilled and healthy than those left in home countries). In Portugal, data on ethnic origin are not collected in the census or in death registrations and in England and Wales they are not collected in death registrations. Country of birth is, therefore, used as a proxy for ethnicity, as is typical in this type of analysis. European ancestry is possible for some of those classified as African migrants, given that many white Portuguese settled in the ex-colonies. This would have weakened the African effect reported here, if it is assumed that white Portuguese classified as African migrants would have a lower mortality risk. The Cape Verde group is least likely to be affected by this bias. In the UK, West Africans are a more established minority with a substantial proportion born in the UK. Finally, census data omit those not legally resident in the country but this group could be included in death registrations, which could inflate the death rates for Africans.

Our findings suggest that the adverse AIDS mortality experience of Africans in Portugal could be linked to socio-environmental factors. Whether some of the excess mortality among Africans could be accounted for by the combination of originating from countries with generalised HIV epidemics and potentially limited access to health services (and, therefore, late diagnosis of HIV) requires further research. Health data on migrants are sparse in Portugal and in England and Wales; there is a need to ensure the inclusion of relevant data items on ethnicity in national monitoring systems. National HIV surveillance should also ensure collection of data on length of time in the country of report along with probable country of acquisition to aid interpretation of mortality rates and help inform HIV prevention efforts.

**Key messages**

- Compared with people born in Portugal, African migrants had higher mortality for infectious diseases including AIDS.
- Infectious disease and AIDS death rates were higher among those who were unmarried than those who were, and among men from manual occupational classes than among non-manual.
- Infectious disease and AIDS death rates in Portugal were much higher than in England and Wales; furthermore, Africans in Portugal also fare worse than Africans in England and Wales.
